# Cancer Stem Cell Markers—Clinical Relevance and Prognostic Value in High-Grade Serous Ovarian Cancer (HGSOC) Based on The Cancer Genome Atlas Analysis

**DOI:** 10.3390/ijms241612746

**Published:** 2023-08-13

**Authors:** Natalia Iżycka, Mikołaj Piotr Zaborowski, Łukasz Ciecierski, Kamila Jaz, Sebastian Szubert, Cezary Miedziarek, Marta Rezler, Kinga Piątek-Bajan, Aneta Synakiewicz, Anna Jankowska, Marek Figlerowicz, Karolina Sterzyńska, Ewa Nowak-Markwitz

**Affiliations:** 1 Department of Gynecology, Obstetrics and Gynecologic Oncology, Division of Gynecologic Oncology, Poznan University of Medical Sciences, Polna 33 St., 60-535 Poznan, Polandsszubert@ump.edu.pl (S.S.);; 2 European Center for Bioinformatics and Genomics, Institute of Bioorganic Chemistry, Polish Academy of Sciences, Noskowskiego 12/14, 61-704 Poznan, Polandmarek.figlerowicz@ibch.poznan.pl (M.F.); 3 Department of Cell Biology, Poznan University of Medical Sciences, Rokietnicka 5D St., 60-806 Poznan, Poland; ajanko@ump.edu.pl; 4 Department of Histology and Embryology, Poznan University of Medical Sciences, Swiecickiego 6 St., 61-781 Poznan, Poland

**Keywords:** epithelial ovarian cancer, HGSOC, TCGA, ovarian cancer prognosis, cancer stem cells, markers, multi-omics, multi-omics data

## Abstract

Cancer stem cells (CSCs) may contribute to an increased risk of recurrence in ovarian cancer (OC). Further research is needed to identify associations between CSC markers and OC patients’ clinical outcomes with greater certainty. If they prove to be correct, in the future, the CSC markers can be used to help predict survival and indicate new therapeutic targets. This study aimed to determine the CSC markers at mRNA and protein levels and their association with clinical presentation, outcome, and risk of recurrence in HGSOC (High-Grade Serous Ovarian Cancer). TCGA (The Cancer Genome Atlas) database with 558 ovarian cancer tumor samples was used for the evaluation of 13 CSC markers (ALDH1A1, CD44, EPCAM, KIT, LGR5, NES, NOTCH3, POU5F1, PROM1, PTTG1, ROR1, SOX9, and THY1). Data on mRNA and protein levels assessed by microarray and mass spectrometry were retrieved from TCGA. Models to predict chemotherapy response and survival were built using multiple variables, including epidemiological data, expression levels, and machine learning methodology. ALDH1A1 and LGR5 mRNA expressions indicated a higher platinum sensitivity (*p* = 3.50 × 10^−3^; *p* = 0.01, respectively). POU5F1 mRNA expression marked platinum-resistant tumors (*p* = 9.43 × 10^−3^). CD44 and EPCAM mRNA expression correlated with longer overall survival (OS) (*p* = 0.043; *p* = 0.039, respectively). THY1 mRNA and protein levels were associated with worse OS (*p* = 0.019; *p* = 0.015, respectively). Disease-free survival (DFS) was positively affected by EPCAM (*p* = 0.004), LGR5 (*p* = 0.018), and CD44 (*p* = 0.012). In the multivariate model based on CSC marker expression, the high-risk group had 9.1 months longer median overall survival than the low-risk group (*p* < 0.001). ALDH1A1, CD44, EPCAM, LGR5, POU5F1, and THY1 levels in OC may be used as prognostic factors for the primary outcome and help predict the treatment response.

## 1. Introduction

High-grade serous ovarian cancer (HGSOC) is a heterogenous malignancy and one of the leading causes of cancer-related deaths in women worldwide [1].

The cancer cell populations derived from a single patient are highly diverse [2,3]. Cancer stem cells (CSCs), the subpopulation of neoplastic cancer cells able to self-renew [4,5], contribute to chemotherapy resistance [2,5,6,7]. CSCs have been detected in OC tumors in the early and advanced stages before and after the conventional treatment [8,9,10]. The presence of CSCs appears to be a feature of all types of OC, including the most heterogenous type—primary HGSOC—independent of its molecular subtypes (mesenchymal, immunoreactive, differentiated, and proliferative) [9,11,12,13,14].

Despite treatment, around 70% of patients experience cancer recurrence [5]. Due to the potential for self-renewal after a long dormancy, CSCs are perceived as the origin of recurrent tumors. Surgery and chemotherapy may eradicate most cancer cells but not the residual population of CSCs [1]. Their self-protection is caused mainly by the slow cell cycle progression, resulting in decelerated metabolism [2,5], which may induce multiple drug resistance mechanisms [3,15]. The persistence of CSCs in tumor tissue and their drug resistance acquired during primary treatment are important factors in tumor relapse after chemotherapy [3,5]. The presence of CSCs has been associated with a worse prognosis in OC [5].

Thus, CSC markers may be valuable prognostic tools in clinical practice [2].

Several CSC markers have been suggested to date. The most often proposed and best-characterized are CD44, CD24, CD117, CD133, and ALDH1A [6]. Their main functions and roles as CSC markers are described in the table below.

**Figure 1:**
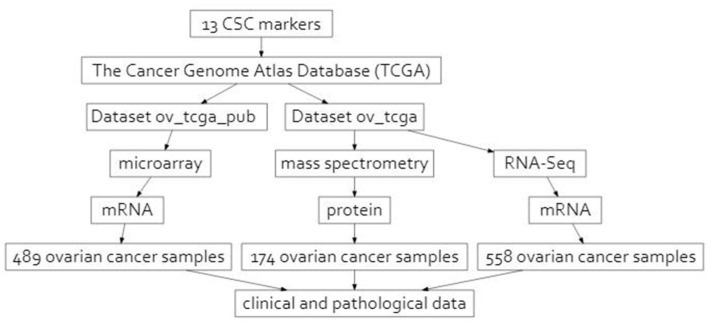
Schematic overview of the study design.

In this study, we aimed to determine the relationships between the clinical features of HGSOC and the expression of selected CSC markers. Besides the well-established CSC markers described above (CD44, CD24, CD117, CD133, and ALDH1A), other proteins might belong to this population. To identify them, we searched for the proteins that were previously mentioned in the literature as potentially related to CSCs in various malignancies, including OC (Table 1). We retrieved proteomic and transcriptomic data and clinical characterizations of 551 HGSOC patients from The Cancer Genome Atlas Database (TCGA). We evaluated the expression levels of the above-defined set of CSC markers (Table 1) in HGSOC (Figure 1). Next, we investigated the relationship of the CSC marker levels with patients’ clinical and pathological features, such as clinical stage, grade of the disease, size of residual disease, following surgery, and responsiveness to chemotherapy. Finally, we assessed the relationship between the analyzed markers and the time to progression and OS (Overall Survival).

## 2. Results

There were 489 patients with complete data for mRNA measured by microarray for 13 CSC markers: ALDH1A1, CD44, EPCAM, KIT, LGR5, NES, NOTCH3, POU5F1, PROM1, PTTG1, ROR1, SOX9, and THY1. Among the analyzed CSC markers, the mass spectrometry data for five proteins (EPCAM, ALDH1A1, CD44, NES, and THY1) were available for 174 patients.

### 2.1. Clinical and Pathological Features

The characteristics of the studied group correspond well to the mean clinical features observed in OC. All the analyzed samples were retrieved from patients suffering from HGSOC. Most patients (71%) in the database labeled “ov_tcga” were at stage IIIC. The mean age at diagnosis was 59.5–60 (interquartile range (IQR): 51–68 amd 51–71 for mRNA and protein samples, respectively). Most OC tissues were characterized as grade 3 (mRNA samples = 82.3%, protein samples = 81.6%). There were no grade 1 tumors.

**Figure A1:**
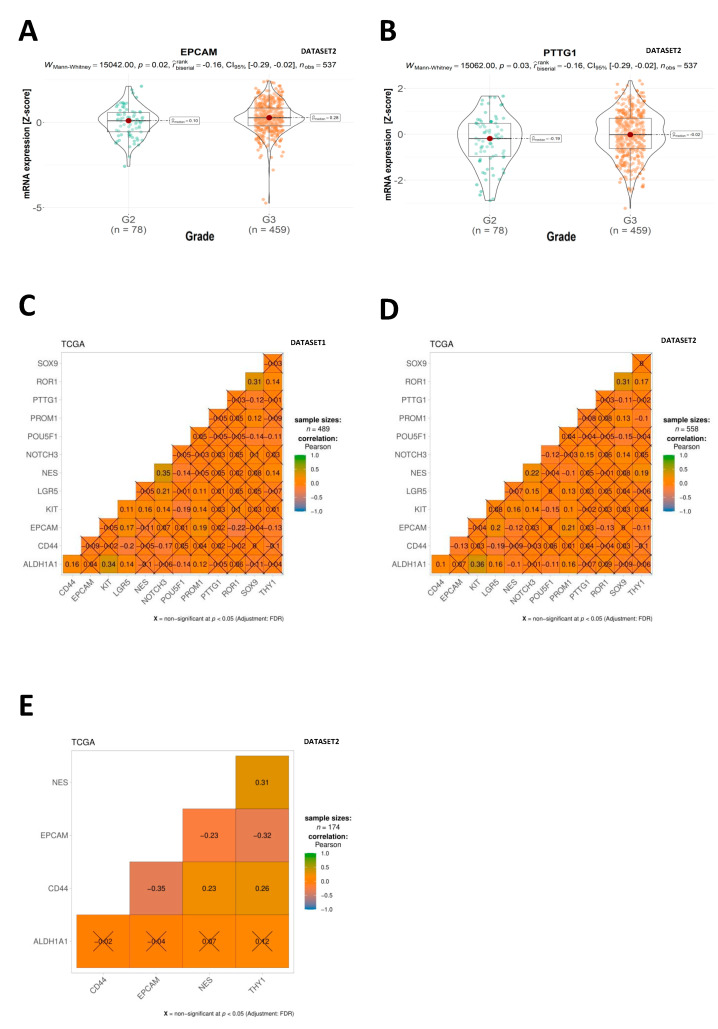
Comparison between clinical and pathological features (**A**,**B**) and correlation matrices (**C**–**E**) (“ov_tcga“ and “ov_tcga_pub“ datasets) (in (**A**), we cut the lower and upper outliers).

The expression of two CSC marker genes was associated with the tumor grade. We found that EPCAM and PTTG1 mRNA levels were higher in grade 3 as compared to grade 2 tumors (“ov_tcga” dataset, *n* = 537, *p* = 0.02; *p* = 0.03, respectively) (Figure A1A and Figure A1B, respectively).

**Figure 2:**
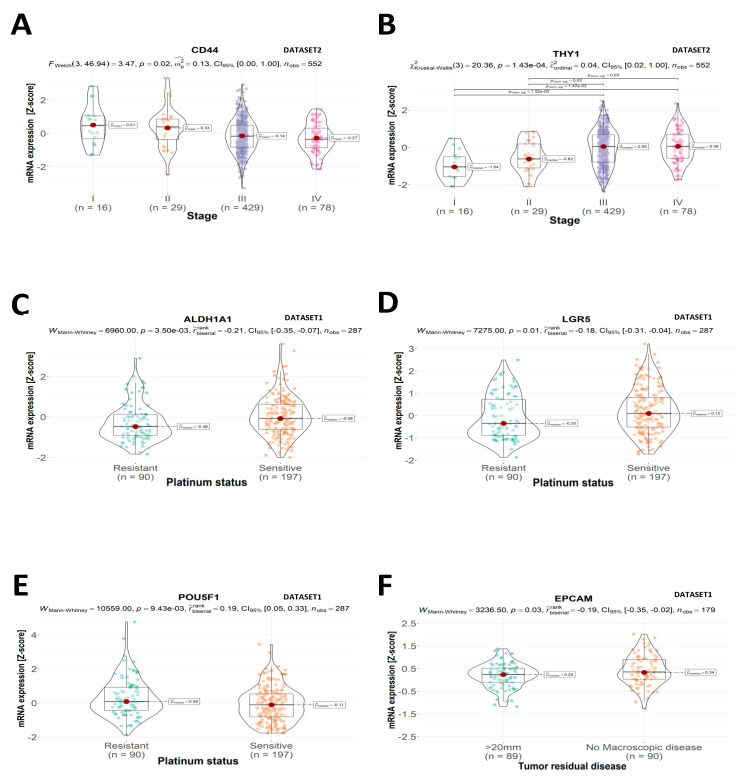
Clinical and pathological features of OC tumors. The expression of CSC markers depending on (**A**,**B**) tumor stage ((**A**) Welch test; (**B**) Kruskal–Wallis test), responsiveness to platinum compounds ((**C**–**E**); Mann–Whitney test), and tumor residual disease following cytoreductive surgery ((**F**); Mann–Whitney test). Values on the Y axis represent mRNA expression as Z scores. Statistical significance and the of statistics used for analysis are described at the top of each plot. The total number of samples is described at the top of each plot. The number of samples in each subgroup is given as “N” (“ov_tcga_pub”—DATASET1 and “ov_tcga”—DATASET2).

The expression of two CSC marker genes was associated with FIGO staging. We found that the CD44 mRNA level was elevated in tumors with FIGO I/II as compared to FIGO III/IV stages (“ov_tcga” dataset, *n* = 552, *p* = 0.02, Figure 2A). At the same time, THY1 mRNA expression was higher in the FIGO III and FIGO IV stages (“ov_tcga” dataset, *n* = 552, *p* = 1.43 × 10^−4^) (Figure 2B).

Furthermore, the expression of CSC genes was linked to the tumor’s platinum sensitivity. We found that higher ALDH1A1 and LGR5 mRNA levels have indicated platinum sensitivity (“ov_tcga_pub” dataset, *n* = 287, *p* = 3.50 × 10^−3^; *p* = 0.01, respectively) (Figure 2C and Figure 2D, respectively). Conversely, mRNA of POU5F1 showed a higher level in platinum-resistant tumors (“ov_tcga_pub” dataset, *n* = 287, *p* = 9.43 × 10^−3^) (Figure 2E).

We also demonstrated that level of one of the CSC markers indicates a chance for complete cytoreduction. The EPCAM level (mRNA) was higher in tumors with no macroscopic residual disease (“ov_tcga_pub” dataset, *n* = 179, *p* = 0.03) (Figure 2F).

### 2.2. CSC Expression Profiles

**Figure 3:**
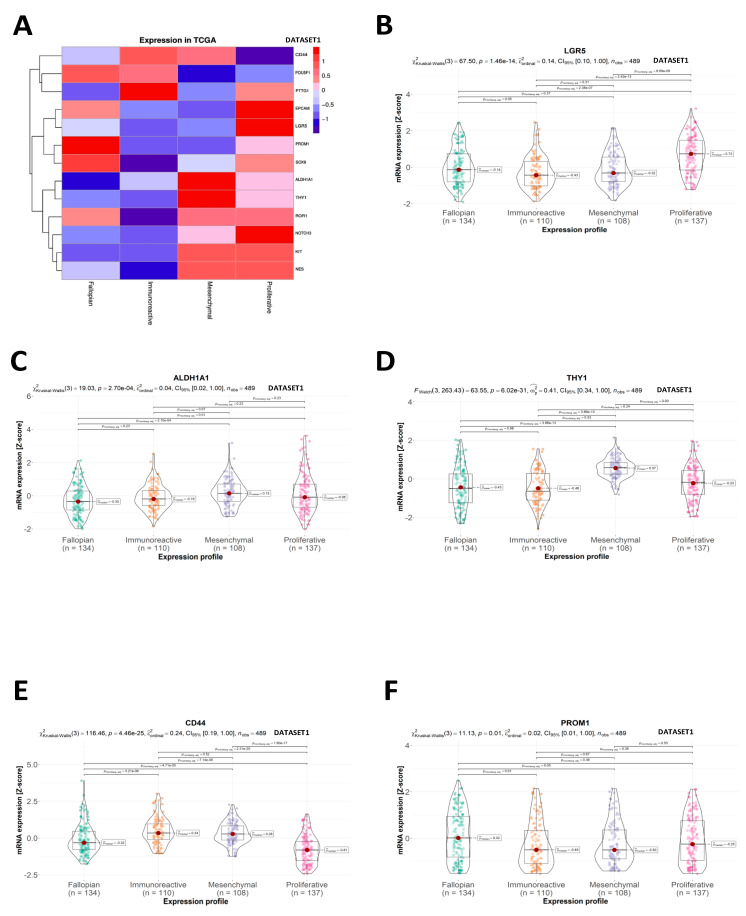
OC expression profiles (“ov_tcga_pub”—DATASET1 and “ov_tcga”—DATASET2). (**A**) normalized expression of CSC markers depending on molecular subtypes of OC (mesenchymal, proliferative, fallopian, and immunoreactive). The colors represent the intensity of expression according to the scale in the top right of the figure. Markers are clustered in order of similarity across their subtypes. (**B**–**F**) mRNA levels represented as Z scores in subgroups according to OC expression profiles. Statistics used: (**B**,**C**,**E**,**F**)—Kruskal–Wallis test; (**D**)—Welch test.

**Figure A2:**
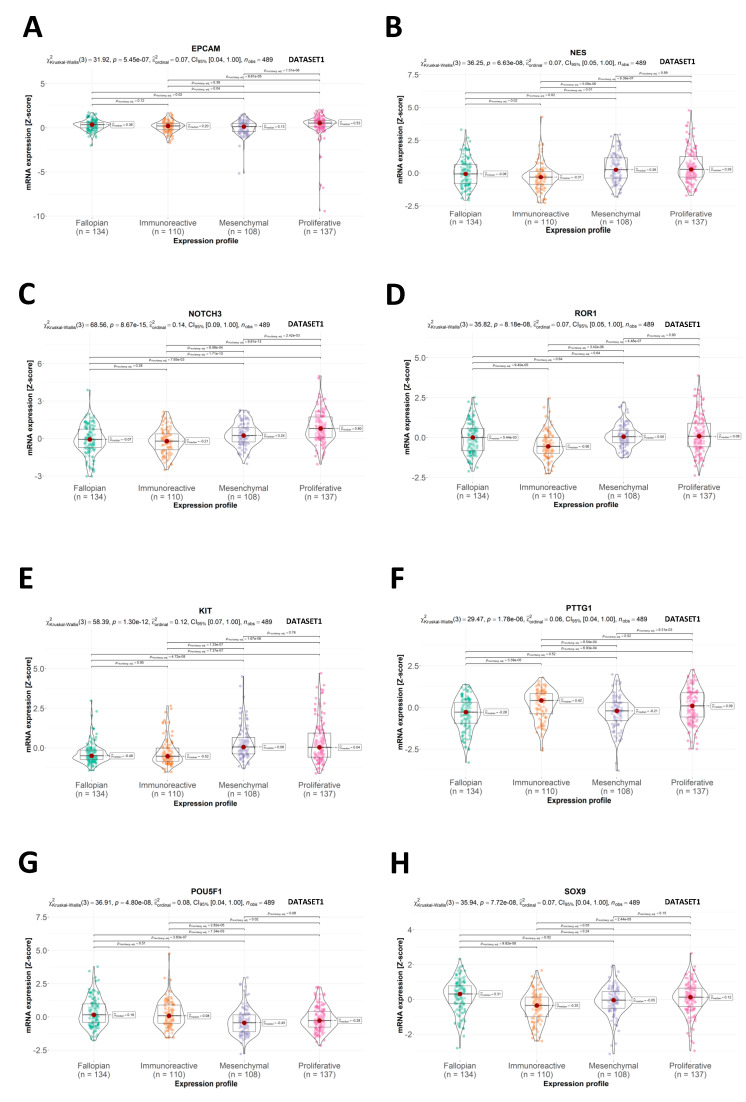
Expression profiles of the “ov_tcga_pub” dataset. mRNA levels are represented as Z scores in subgroups according to OC expression profiles. High amounts of EpCAM (**A**), NES (**B**) NOTCH3 (**C**), ROR 1 (**D**) transcripts were associated with proliferiative profile. The upregulation of KIT (**E**) was associated with mesenchymal profile. Elevated level of PTTG1 (**F**) was associated with immunoreactive profile. The fallopian profile was characterized by elevation of POU5F1 (**G**) and SOX9 (**H**).

Four molecular subtypes of OC are distinguished based on the gene expression pattern [9]. We found that specific CSC markers were linked to OC expression profiles. (Figure 3A). High amounts of EPCAM, NES, NOTCH3, ROR1, and LGR5 transcripts were associated with proliferative profile (“ov_tcga_pub” dataset, Figure A2A–D and Figure 3B, respectively). The mesenchymal profile was characterized by upregulation in ALDH1A1, KIT, and THY1 mRNA (Figure 3C, Figure A2E, and Figure 3D, respectively). The immunoreactive profile was marked by high CD44 and PTTG1 expression (Figure 3E and Figure A2F, respectively). POU5F1, PROM1, and SOX9 gene expressions were more prevalent in the fallopian profile (Figure A2G, Figure 3F, and Figure A2H, respectively).

### 2.3. Overall Survival

**Figure 4:**
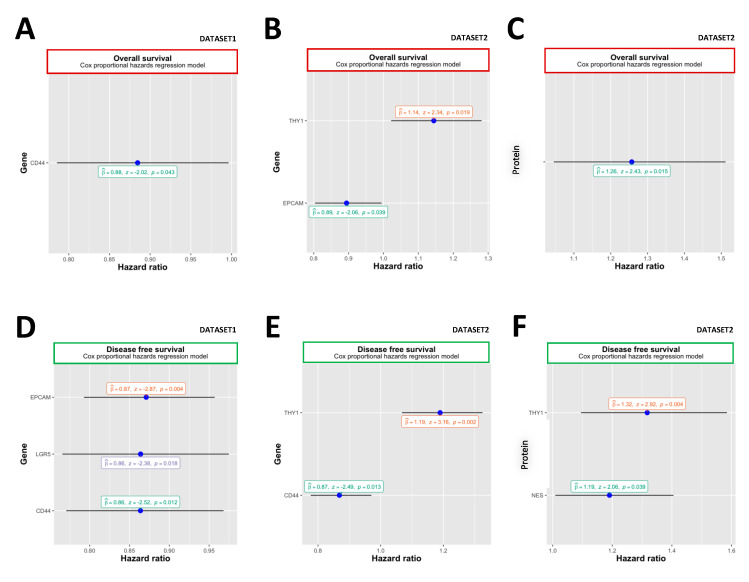
The OS and DFS (“ov_tcga” and “ov_tcga_pub” datasets). Relationship between CSC markers and patient survival. Association between mRNA (miRNA) expression of CSC markers and (**A**,**B**) OS, as well as between DFS (**D**,**E**) based on the “ov_tcga_pub” and “ov_tcga” datasets. Association between protein expression of CSC markers (mass spectrometry) and (**C**) OS and (**F**) DFS based on “ov_tcga” datasets. Panels represent only statistically significant (*p* < 0.05) relationships. Beta coefficient and *p* values of univariate Cox proportional hazard regression models are given in the panels.

We found that the expression of CSC marker genes was associated with OS. In the microarray-assessed dataset, CD44 mRNA levels were associated with longer OS (dataset “ov_tcga_pub”, HR = 0.88, 95%, 0.79–1.00; *p* = 0.043) (Figure 4A). The beneficial effect was also observed in the “ov_tcga” dataset, where EPCAM mRNA expression predicted longer OS (HR = 0.89, 95%, 0.80–0.99; *p* = 0.039, Figure 4B), while THY1 mRNA level was associated with poorer OS (HR = 1.14, 95%, 1.02–1.28; *p* = 0.019) (Figure 4B). Consistently, the level of THY1 protein was also associated with shortened OS (“ov_tcga” protein dataset, HR = 1.26, 95%, 1.05–1.51; *p* = 0.015) (Figure 4C).

### 2.4. Disease-Free Survival

The expression of the EPCAM gene was associated with longer DFS (“ov_tcga_pub” dataset, HR = 0.87, 95%, 0.79–0.96; *p* = 0.004), LGR5 (HR = 0.86 95%, 0.77–0.97; *p* = 0.018) and CD44 (HR = 0.86, 95%, 0.77–0.97; *p* = 0.012) (Figure 4D). Consistently, a higher amount of CD44 transcripts was associated with longer DFS (dataset “ov_tcga”, HR = 0.87, 95%, 0.80–1.01; *p* = 0.013) (Figure 4E). In contrast, THY1 expression predicted reduced DFS (HR = 1.19, 95%, 1.07–1.33; *p* = 0.002) (Figure 4E). In agreement, the levels of THY1 (“ov_tcga” dataset, HR = 1.32, 95%, 1.09–1.58; *p* = 0.004) and NES (“ov_tcga” dataset, HR = 1.19, 95%, 1.01–1.41; *p* = 0.039) proteins were associated with poorer DFS (Figure 4F).

### 2.5. Correlation Analysis

In both datasets, we found significant positive correlations between ALDH1A1 and KIT mRNA levels (r = 0.34; r = 0.36, *n* = 489; *n* = 558, *p* < 0.05, “ov_tcga_pub” and “ov_tcga” datasets, respectively; Figure A1C and Figure A1D, respectively) and between ROR1 and SOX9 (r = 0.31; r = 0.31, *n* = 489; *n* = 558, *p* < 0.05, “ov_tcga_pub” and “ov_tcga” datasets, respectively (Figure A1C and Figure A1D, respectively). Furthermore, in the “ov_tcga_pub” dataset, NOTCH3 expression correlated well with NES (“ov_tcga_pub”, r = 0.35, *n* = 489, *p* < 0.05, Figure A1C).

We found a negative correlation between the EPCAM and THY1 proteins and between the EPCAM and CD44 proteins (dataset “ov_tcga”, r = −0.32; r = −0.35, *n* = 174, *p* < 0.05) (Figure A1E). We also demonstrated that the THY1 protein level was increased in association with NES protein (“ov_tcga”, r = 0.31, *n* = 174, *p* < 0.05) (Figure A1E).

### 2.6. Multivariate Predictive Analysis

To verify the collectively predictive power of 13 CSC markers with respect to the carboplatin treatment outcome and patients survival, we performed multivariate predictive analysis using machine learning (ML) methods. The importance of the CSC markers for the ML models’ prediction was assessed jointly with the clinical and pathological features. We estimated the performance of each ML model to verify whether they learn from the data that might be useful for prospective studies.

**Figure 5:**
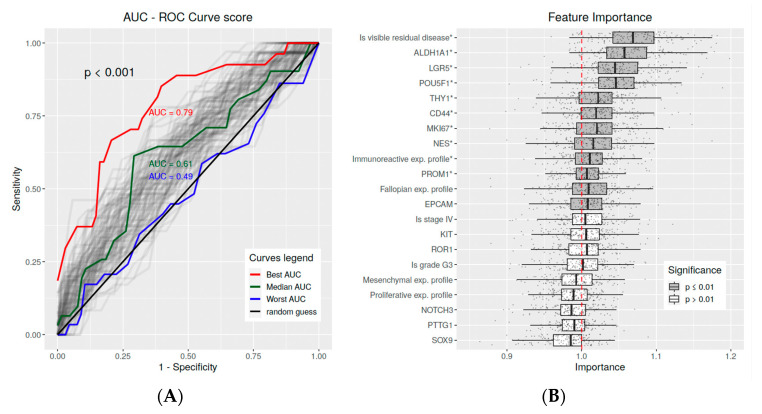
Results of the carboplatin therapy outcome predictive analysis. (**A**) Expected outcome prediction performance of the random forest classifier trained on the “ov_tcga_pub” dataset. The performance was estimated using repeated fivefold cross-validation (CV) and the AUC score. The median AUC = 0.61 95%, 0.601–0.63, which is greater than a random guess with *t*-test(μ_0_ = 0.5) = 22.087, *p* < 0.001. (**B**) Importance of the “ov_tcga_pub” dataset features for therapy outcome classification was estimated using the permutation feature importance score measured using the AUC score and the classifiers obtained from the CV. (*) The significance of the importance measurements was tested with a one-sample Wilcoxon test under the alternative hypothesis, i.e., that the importance mean > 1.0, *p* ≤ 0.001.

**Figure A3:**
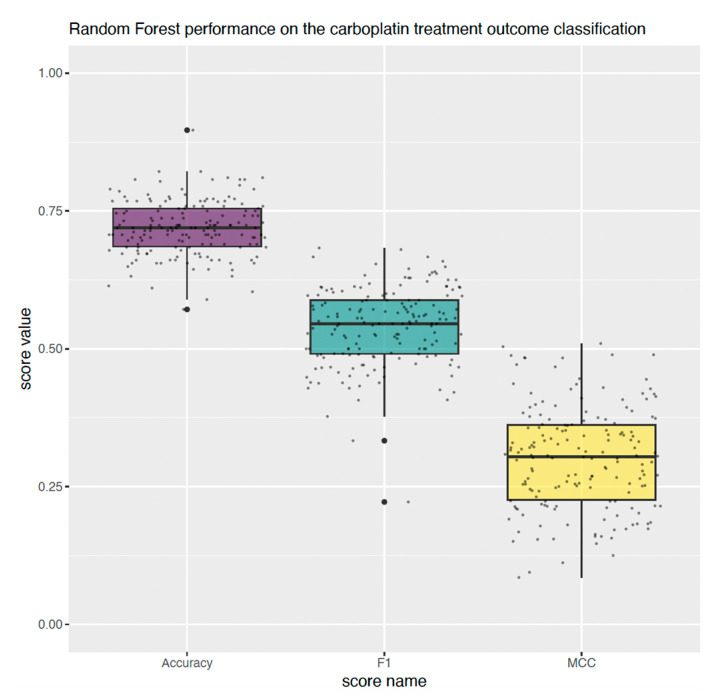
Expected performance of the random forest classifier trained on the “ov_tcga_pub“ dataset estimated using repeated fivefold cross validation and different machine learning model assessment scores, including accuracy, F1 score, and MCC (Matthews correlation coefficient).

For the predictive analysis of carboplatin treatment outcome, the random forest classifier performance on the “ov_tcga_pub” microarray dataset (*n* = 287) was estimated using the repeated fivefold cross-validation method (CV) and the AUC (area under the ROC curve) score. The expected performance of the model is significantly higher than the efficacy of a random guess (*t*-test(μ_0_ = 0.5) = 22.964, *p* < 0.001), with a median estimated AUC = 0.61, 95%, 0.601–0.63 (Figure 5A). We also calculated additional model performance scores with the following median values: accuracy = 0.71, 95%, 0.702–0.714, F1 = 0.53, 95%, 0.525–0.545, and MCC = 0.28, 95%, 0.266–0.293 (Figure A3).

**Figure 6:**
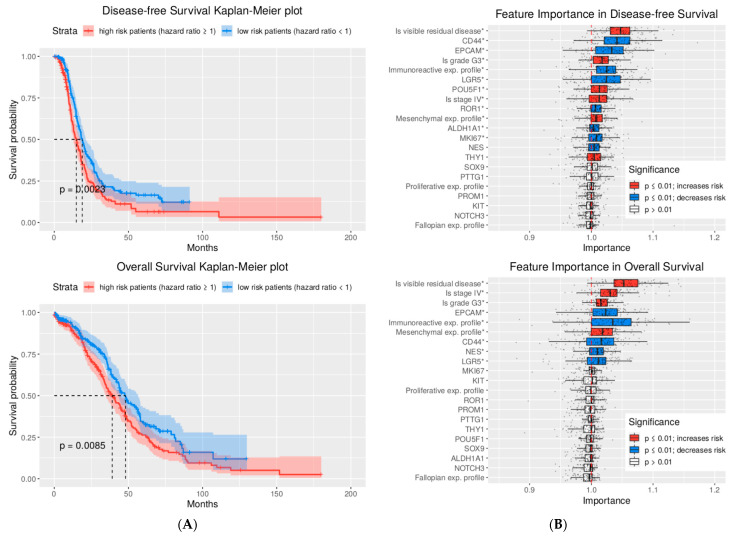
Results of disease-free and overall survival multivariate analyses. (**A**) Expected stratification capability of the Cox proportional hazards models with ridge regularization fitted to the “ov_tcga_pub” dataset. The stratification performance was estimated using repeated fivefold cross-validation (CV) and c-index score. The null hypothesis of the log-rank test that the low-risk and high-risk strata have identical hazard functions was rejected, with *p* < 0.0023 and *p* < 0.0085 for the disease-free and overall survival data, respectively. (**B**) Importance of the “ov_tcga_pub“ dataset features for disease-free and overall survival hazards risk prediction was estimated using the permutation feature importance score measured using the c-index score and the models obtained from the CV. The significance of the importance measurements was tested with a one-sample Wilcoxon test under the alternative hypothesis, i.e., that the importance mean > 1.0. (*) The aterisk indicates features for which an estimated importance mean is >1.0 with *p* ≤ 0.001.

**Figure A4:**
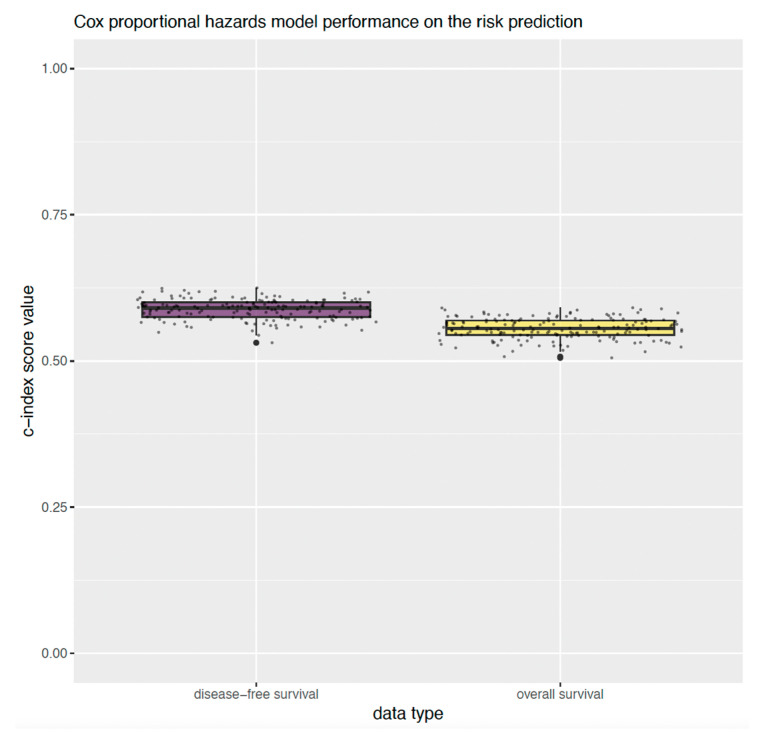
Expected risk prediction performance of the Cox proportional hazards models with ridge regularization fitted to the “ov_tcga_pub“ dataset disease-free and overall survival data. The performance was estimated using repeated fivefold cross validation (CV) and the c-index score.

For the DFS and OS analysis, we estimated the performance of Cox proportional hazards models trained on the “ov_tcga_pub” microarray dataset (*n *= 487) with ridge regularization. The performance was assessed using the c-index score measured during repeated fivefold cross validation. The median equated c index = 0.59, 95%, 0.585–0.59 (*t*-test(μ_0_ = 0.5) = 73.94, *p* < 0.001) for the DFS, and c index = 0.56, 95%, 0.555–0.56 (*t*-test(μ_0_ = 0.5) = 36.76, *p* < 0.001) for OS (Figure A4). Additionally, the stratification capabilities of the models were estimated by dividing patients into low- and high-risk groups using the calculated median hazard risks of recurrence or death of the patients based on CSC markers. For DFS, the low- and high-risk groups were determined to be significantly divergent (*p* < 0.002), with 4.72 months of difference between the times of the strata median survival probabilities. In the case of OS, the low- and high-risk groups were separated (*p* < 0.001) by 9.12 months in median survival times (Figure 6A).

Knowing that all the trained models are expected to be relevant, we assessed the importance of the “ov_tcga_pub” dataset features using the permutation feature importance (PFI) score (Figure 5B and Figure 6B). The most important predictive factor of the carboplatin therapy outcome, DFS, and OS was the presence of residual disease, which increases the risk of both disease recurrence and patient death (*p* < 0.001). Another significant pathological feature was the immunoreactive profile of gene expression, which was also meaningful for carboplatin outcome prediction and decreased the risks for both OS and DFS. The pathological features important for predicting the DFS and OS hazard risks included mesenchymal expression profile of gene expression, FIGO stage IV, tumor grade 3, and MKI67 proliferation marker expression (*p* < 0.001).

We subsequently analyzed the PFI scores of the 13 considered CSC markers. We report that the CD44, LGR5, NES, and EPCAM CSC markers were significantly important outcome predictors (*p* < 0.01) in all multivariate analyses. Also, the POU5F1, THY-1, and ALDH1A1 markers were determined as important for both the carboplatin therapy outcome binary classification and hazards risks assessment. Interestingly, only the POU5F1 and THY1 markers were defined as the risk-increasing factors of DFS. The rest of the listed markers were described as risk-decreasing factors.

## 3. Discussion

Among all the analyzed CSC markers, our study shows that the expression of six genes—CD44, ALDH1A1, EpCAM, THY-1, POU5F1, and LGR5—is significant in OC in terms of clinical outcome, including stage and grade of the disease, as well as the platinum sensitivity and patient survival.

We found that the expression of the CD44 gene was associated with lower FIGO stage and, hence, better OS and DFS of ovarian cancer patients. Moreover, we observed higher levels of the CD44 gene in the immunoreactive subtype of OC. This type is characterized by a more favorable prognosis and extensive intratumoral T-cell infiltration [17]. In fact, CD 44 might play an essential role in regulating the lymphocyte infiltration process in OC tumor tissue, as it is a molecule known for its role in lymphocyte activation and homing [33]. Our results are consistent with those of Sillanpaa S et al. [34] and Sosulski et al. [35] and show the association between CD44 gene expression, lower FIGO stage, and better survival times of OC patients. Still, the role played by CD44 in carcinogenesis is more complicated. In some reports, the increase in CD44 gene expression was indicated as a prognostic factor of both shorter OS and DFS of OC patients. Its correlation with higher FIGO stage and grade was also demonstrated [36,37]. The observed differences in CD44 impact on OC biology might be caused by many mRNA splice variants of CD44 and their various effects on the tumor characteristics [35,37]. As a cell-surface glycoprotein, it undergoes numerous post-translational modifications, which may distort its initial abundance and function.

Our results based on the analysis of ALDH1A1 gene expression in OC show that it is highest in the mesenchymal type of OC, which is considered the most aggressive subtype [38]. A negative correlation was demonstrated between an increased number of ALDH1A1-positive cells and patient survival [39,40]. Given many discordant reports, a meta-analysis of ALDH1A function was performed by Ruscito et al., revealing that high levels of ALDH1A1 protein correlated with worse OS and DFS of OC patients [41]. However, our study also revealed that the accumulation of mRNA for ALDH1A1 in tumor tissue is correlated with higher sensitivity to platinum-based chemotherapy, which is usually an indicator of a better prognosis for OC patients. ALDH1A1 may have an impact on chemoresistance. A broad analysis of multiple ovarian cancer cell lines revealed significantly higher ALDH1A1 gene expression in taxane- and platinum-resistant cell lines [42,43]. ALDH1A1 is also active in platinum-resistant cancer cells residing in hypoxic regions [9,40]. In several studies in other cancer types, including breast cancer, stromal ALDH1A1 protein level, as measured by immunohistochemistry, was associated with better clinical outcomes [44].

Our detailed evaluation of TCGA data revealed that a higher level of EPCAM mRNA was associated with a higher percentage of optimal debulking during primary surgery. Furthermore, EPCAM is among the genes whose expression is associated with a positive response to platinum compounds. It is also related to improving DFS and OS survival in OC patients [45]. Thus, the data from the human protein [46], together with the results of our study, indicate that EpCAM gene expression is associated with a favorable prognosis of OC patients. Our univariate and multivariate analyses demonstrated that the EpCAM mRNA level is associated with longer PFS and OS.

Interestingly, we revealed that improved patient survival times are correlated only with increased EpCAM mRNA levels. Such a link was not observed at the protein level. Previous studies showed [27,45] the correlation between EpCAM protein level and decreased OS and higher FIGO stage and grade of the disease [27]. Thus, the EpCAM protein was proposed as a significant factor contributing to the chemoresistance of OC cells. All this suggests that EPCAM is an important marker of OC prognosis. However, further research is needed to completely understand its role in the disease.

Our analysis consistently demonstrated a correlation between THY-1 gene expression and the unfavorable prognosis of OC patients. We have shown that THY-1 mRNA and protein levels are considered an independent factor associated with poor OS, with higher levels in FIGO stage III and IV and the mesenchymal subtype of OC. Moreover, THY-1 mRNA but not protein level was associated with platinum resistance and poor DFS in OC. THY1 transcriptional activity was associated with the highly invasive and metastatic potential of OC cells, and its correlations with significantly shorter median DFS and OS in patients with HGSOC were previously demonstrated [26,47]. However, depending on the cancer type, THY-1 might have ambivalent properties regarding its anti- or protumoral activities. In some studies on OC, THY-1 was suggested to have a tumor-suppressive role [24,48,49]. The mechanism by which the THY-1 gene may inhibit OC cell growth is still unclear. Its ambivalent impact reported in many studies may be the result of its presence in immune and cancer cells [50]. However, among all markers we have studied, THY-1 has a consistently unfavorable impact on the protein and mRNA levels. Therefore, it is an interesting candidate for further studies.

Another cancer stem cell marker with a substantial impact on the platinum sensitivity of OC cells was POU5F1. Our study revealed that its high expression at the mRNA level was associated with platinum resistance and, therefore, worse DFS in OC patients. In a study by Xie W et al. [51], the POU5F1 protein level was significantly correlated with higher tumor grades and lymph node metastases. Therefore, it was suggested to promote proliferation and metastasis in OC. It was also considered an independent predictor of poor prognosis and progression of OC [51]. Moreover, a study by Ruan Z et al. [52] suggested the significant role of POU5F1 in stemness and drug resistance of OC. It was also correlated with a more aggressive phenotype of OC.

LGR5 is another CSC marker presented as a predictor of good response to platinum therapy. Our multivariate and univariate analysis showed that LGR5 was associated with longer DFS. We also observed that the LGR gene was associated with the proliferative type of OC. The expression of LGR was previously shown to be associated with higher stages of the disease, and its upregulation was associated with metastases in OC patients [28]. The increased expression of LGR5 markedly promotes the growth and the EMT of ovarian cancer cells. Thus, it may promote tumorigenesis and the formation of metastases. Though we observed higher expression in the proliferative subtype, samples more responsive to platinum also had higher levels of LGR5 mRNA. Consistently with our results, Kim and colleagues [53] revealed that in HGSOC, high LGR5 expression was associated with improved PFS.

In this study, we analyzed data on the expression of a set of CSC markers in TCGA. Thanks to this approach, we validated specific observations regarding well-known markers and studied proteins of unclear relevance. TCGA is a database containing a single tumor sample that does not provide insight into the heterogeneity of HGSOC. It must be considered as a limitation when interpreting the results. However, it may serve as a valuable tool for screening and narrowing a set of CSC markers in terms of more detailed analyses. Consequently, some proteins we included have well-established CSC marker status, whereas others are still considered candidates.

## 4. Materials and Methods

### 4.1. Data Retrieval

Transcriptome (microarray and RNA sequencing) data were obtained from TCGA using http://www.cbioportal.org/public-portal/ (accessed on 1 September 2022) as CGDS object (mycgds) compatible with downstream analysis with R programming [54].

For analysis of microarray data from OC tumors, values provided in the genetic profile “ov_tcga_pub_mrna_median_Zscores” in the study labeled as “ov_tcga_pub” were selected [55]. We analyzed patients included in the “ov_tcga_pub_all” case list. Patients with missing values or genes defined only in a subset of patients were excluded from the analysis. For comparison of RNA-Seq and microarray input, OC tumor values provided in the genetic profile “ov_tcga_rna_seq_v2_mrna_median_Zscores” in the study labeled as “ov_tcga” were selected. We used clinical characterization of the patients obtained by the function cgdsr::getClinicalData(). The predefined OC expression profiles (fallopian, immunoreactive, mesenchymal, and proliferative) were retrieved with the function cgdsr::getCaseLists(mycgds,‘ov_tcga_pub’). The cgdsr::getCaseLists (mycgds,‘ov_tcga_pub’) function allows the user to fetch a list IDs of the patients from the 0v_tcga_pub study. The predefined OC expression profiles (fallopian, immunoreactive, mesenchymal, and proliferative) could be retrieved with the cgdsr::getProfikeData function.

We are aware that there is an overlap between the “ov_tcga_pub” and “ov_tcga” datasets. Given that the samples have different categories of clinical annotation (such as response to platinum therapy or pathological features of the tumor), we analyzed both sets. All data processing was performed in the R programming language (version 4.2.1) using RStudio (version 2022.12.0 + 353 “Elsbeth Geranium” Release).

### 4.2. Clinical and Pathological Features

In statistical and predictive analyses, we analyzed mRNA expression assessed by a microarray from the study labeled “ov_tcga_pub” (*n* = 489) for 13 CSC markers: ALDH1A1, CD44, EPCAM, KIT, LGR5, NES, NOTCH3, POU5F1, PROM1, PTTG1, ROR1, SOX9, and THY1. Data for the remaining four out of 16 CSC markers (c-Kit, SNORD89, SNORA72, and TMSB4X) were incomplete and therefore excluded from further analysis. We compared the markers’ expression with the clinical and pathological features, including DFS, OS, grade, platinum sensitivity status, primary therapy outcome, tumor residual disease, stage, and expression profile.

For some patients, only data on mRNA expression were available, whereas for others, data on both mRNA and protein expression were available. Additional statistical analyses were performed on the dataset labeled as “ov_tcga”. This included analysis of mRNA (*n* = 558) expression data of the same 13 CSC markers described above and mass spectrometry measurements of the 5 proteins (*n* = 174) produced by the ALDH1A1, CD44, EPCAM, NES, and THY1 genes. Only a part of the dataset records contained information about both mRNA and protein levels; the rest comprised only mRNA data. We statistically compared the expression of the mRNA with clinical and pathological features, including clinical stage, DFS, OS, grade, histological type, treatment outcome, and expression profile. According to TCGA database, after therapy, platinum-resistant cancer recurs within six months, whereas in platinum-sensitive cancer, the recurrence takes place more than six months after completion of platinum-based chemotherapy. In our statistical and predictive analyses, we included information about MKI67 as a proliferation marker.

### 4.3. Data Preprocessing

For predictive analysis of carboplatin therapy outcome and multivariate survival analyses, we conducted additional data preprocessing steps. First, we excluded records with values of categorical features that were highly under-represented (<10 records). Less under-represented values of ordinal categorical features were grouped with more numerous values in the sequence. For multivariate analysis, the residual disease feature was grouped into categories: “no visible residual disease” and “visible residual disease” (tumor tissue of any size remaining after the surgery). Tumor grade was divided into “grade G3” and “non-grade G3” (G1 and G2) groups, and cancer stage features were split into “stage IV” and “non-stage IV” groups.

To facilitate the use of machine learning methods, we performed numerical encoding of categorical features. The expression profile feature was one-hot-encoded as four separate variables corresponding to either immunoreactive, fallopian, mesenchymal, or proliferative expression profiles. Moreover, we performed binary encoding on categorical variables that were previously divided into two groups. In such a way, we encoded residual disease assessment as “Is visible residual disease” binary feature, tumor grade as “Is grade G3”, and cancer stage as “Is stage IV”.

Lastly, for the purpose of training carboplatin therapy outcome classifiers, we tackled the class imbalance issue with random oversampling. We did not apply any class balancing methods for either of the survival analyses, as they provide no performance gains to the models trained on censored survival data [56].

### 4.4. Statistics

In our statistical analyses, we tested the normality of the data distribution using a Shapiro–Wilk test. Student’s *t*-test (t.test) was used for data with a normal distribution to calculate the statistical significance (*p*-value). A Mann–Whitney *U* test was used for data with a non-Gaussian distribution. Kruskal–Wallis and ANOVA tests were used to compare the data of more than two independent groups for non-normally and normally distributed data, respectively. The R package ‘ggstatsplot’ was used to generate plots [57].

The data were presented as boxplots with an interquartile range (IQR), median, minimum, and maximum values. Jitter plots were used to demonstrate the scattering of the data. The central line in the plots corresponds to the median. The upper and lower “hinges” correspond to the first and third quartiles, respectively. Plot whiskers extend to the most extreme data point, which is within 1.5 times the interquartile range from the box. We visualized data representing individual patients as dots in the boxplots. The correlations were calculated using Pearson’s correlation and presented in a correlation matrix with Pearson correlation coefficients (r) and *p* values using the ggstatsplot::ggcorrmat() function.

### 4.5. Univariate Analysis of Overall and Disease-Free Survival

The relationships between OS and DFS and expression data were analyzed based on a study labeled “ov_tcga_pub”, as well as mRNA and mass spectrometry data from a study labeled “ov_tcga”. We applied the survival and survminer libraries. A Cox proportional hazards regression model was used to perform survival analysis. The data were presented as hazard ratios with 95% confidence intervals, beta (β) coefficients, and calculated *p* values. The plots were generated by the ggstatsplot::ggcoefstats() function.

### 4.6. Predictive Analysis of Carboplatin Treatment Outcome

We assessed the predictive power of a random forest model that was trained on the “ov_tcga_pub” microarray dataset to perform binary classification platinum treatment efficacy (*n* = 287). The features considered in analysis are expression levels of the same 13 CSC markers that were used for the univariate analysis. Moreover, the MKI67 proliferation marker, residual disease assessment, tissue expression profile, tumor grade, and cancer stage features were included.

To create random forest models, we used the randomForest::randomForest function [58]. We assessed the performance of classifiers using AUC score and a 5-fold cross validation (CV) method repeated 34 times, which resulted in 170 models trained for performance estimation. We optimized each model’s hyperparameters using 5-fold cross validation and a grid search performed on its training set. The hyperparameters were also evaluated using the AUC score. For each model, we chose parameters that showed the best performance on validation sets. Random forest models were optimized for the best values of forest size in a range of 100–300 and tree node size in a range of 5–30. To test whether the estimated performance was significantly better than a random guess, we performed a one-sample Student’s *t*-test (distribution passed the Shapiro–Wilk normality test) and checked an alternative hypothesis, i.e., that the AUC score distribution > 0.5. The AUC score was calculated using the pracma::trapz function [59]. The ROC plot was prepared using the ggplot2 package. Each plotted curve represents a performance assessment of one of the models trained during CV.

### 4.7. Multivariate Analysis of Overall and Disease-Free Survival

We used the Cox proportional hazards model with ridge regularization [60] to analyze OS and DFS of patients in the “ov_tcga_pub” microarray dataset. We considered only records with complete mRNA expression levels and complete clinical and pathological data for the 13 CSCs, including residual disease assessment, tissue expression profile, tumor grade, and cancer stage information (*n* = 487). We included the MKI67 proliferation marker in the analysis.

The models were obtained using the glmnet::cv.glmnet function [61,62]. For ridge regularization, we set the alpha parameter to zero. Moreover, no baseline function was assumed, as we focused only on hazard risk prediction. We assessed the performance of the hazard models trained on the “ov_tcga_pub” dataset using the c-index score and a 5-fold cross-validation (CV) method repeated 34 times, which resulted in training 170 models for performance estimation. The cv.glmnet function incorporates a hyperparameter optimization method by cross validation. The optimization was performed on each model’s training set with 5-folds CV, with the c index as a validation score. The optimized parameter was lambda, which determines the strength of the regularization. Other parameters were left at default values. To test whether assessed performance was significantly better than a random guess, we performed a one-sample Student’s t-test (distribution passed the Shapiro–Wilk normality test) and checked an alternative hypothesis, i.e., that the c index > 0.5.

To further assess the performance of the hazards model trained on the “ov_tcga_pub” dataset, we stratified patient risk into low- (patient median risk <1.0) and high-risk (patient median risk ≥ 1.0) strata. The efficacy of the patient division was determined using a log-rank test. We visualized the stratification results with Kaplan–Meier survival curves calculated using the survival::survfit function and drawn with the survminer::ggsurvplot function [63]. The curves were additionally described with confidence intervals. The dashed lines indicate the time of median survival probability for each stratum.

### 4.8. Feature Importance Analysis

To uniformly estimate the importance of “ov_tcga_pub” dataset features, in all our machine learning analyses, we used the permutation feature importance (PFI) model-agnostic score [64]. The PFI is measured as an increase in the model’s prediction error after permuting the feature’s values. The PFI scores were obtained using the iml::FeatureImp$new function with “ratio” as a comparison method [65].

For PFI measurements, we used the same models that were previously trained for the purpose of performance estimation. PFI measurement was repeated 30 times on each model and its corresponding test set, resulting in 5100 measurements per feature in total. To test whether a feature was significantly important, we performed a one-sample Wilcoxon test (distributions did not pass the Shapiro–Wilk normality test) and checked an alternative hypothesis, i.e., that the obtained PFI score distribution is greater than the value, indicating no prediction importance (PFI > 1). A feature was determined as important if its estimated PFI was higher than 1 with *p* < 0.01.

The feature importance is presented as a box plot with interquartile range, median, and minimum and maximum values drawn in the same way as in our statistical analysis using the ggplot2 package (version 3.4.2).

## 5. Conclusions

Our study showed that the expression of six CSC markers (CD44, ALDH1A1, EpCAM, THY-1, POU5F1, and LGR5) is significant in OC in terms of clinical outcome, including stage and grade of the disease, as well as the platinum sensitivity and patient survival. The CD44, ALDH1A1, EpCAM, THY-1, POU5F1, and LGR5 levels in OC may be used as prognostic factors for the primary outcome and may be beneficial in predicting the treatment response.

## Figures and Tables

**Table 1:** CSC markers selected based on a review of the literature. The function and potential contribution to the phenotype of cancer stem cells are described following the given reference.

CSC Marker	Full Name	Function	References
ALDH1A1	Aldehyde Dehydrogenase 1 Family Member A1	Enzyme belonging to the aldehyde dehydrogenase family of proteins participating in the biosynthesis of retinoic acid and allowing for the regulation of proper proliferation and differentiation of cancer stem cells.	[16]
CD44	CD44 molecule	A cell-surface glycoprotein that promotes metastasis, stem cell-like phenotypes, and chemoresistance.	[17]
PROM1 (CD133)	Prominin-1	Transmembrane protein that promotes stemness and strengthens adhesion and clearance of mesothelial cells; it causes an increase in peritoneal adhesion. It entails improved adherence to the metastatic niche and infiltration of the peritoneal tissue during metastases.	[18,19]
c-Kit (CD117)	KIT proto-oncogene receptor tyrosine kinase	This cytokine receptor is closely related to the neovascularization of epithelial OC tissue and the formation of vasculogenic mimicry. CSCs are associated with the formation of vasculogenic mimicry, which therefore acts as a molecular CSC marker.	[20]
POU5F1 (OCT4)	POU class 5 homeobox 1 (octamer-binding transcription factor 4)	It encodes embryonic transcription factors that are vital for quiescence, pluripotency, and long-term self-renewal—properties that are characteristic of CSCs.	[21]
NOTCH3	Notch Receptor 3	A protein whose activation increases the adhesion between ovarian tumor cells and collagen-rich peritoneal surfaces.	[22]
SOX9	SRY-box transcription factor 9	A protein that regulates apoptotic and proliferative properties. It allows OC to survive in hypoxic conditions.	[23]
SNORD89	Small nucleolar RNA, C/D box 89	A small nucleolar RNA that promotes stem-cell-like characteristics via the Notch1/c-Myc pathway.	[23,24,25]
SNORA72	Small nucleolar RNA, H/ACA box 72		
Thy-1 (CD90)	Thy-1 cell surface antigen	GPI-anchored protein located on the cell surface correlated with increased self-renewal and proliferative ability of OC cells. It acts as a tumor suppressor in OC. Thy-1 is overexpressed in CSCs.	[23,24,25]
EpCAM	Epithelial cell adhesion molecule	Transmembrane glycoprotein mediating Ca2+-independent homotypic cell–cell adhesion in epithelia. EpCAM regulates chemoresistance by activating the PI3K/Akt/mTOR signaling pathway.	[26]
LGR5	Leucine-rich repeat containing G protein-coupled receptor 5	Protein that may promote epithelial OC development through regulation of the Notch1 signaling pathway, which is associated with CSC self-renewal and drug resistance.	[27]
PTTG1 (Securin)	PTTG1 regulator of sister chromatid separation, securin	Protein with the ability to regulate CSC-associated self-renewal and epithelial–mesenchymal transition pathways.	[28]
ROR1	Receptor tyrosine kinase like orphan receptor 1	A surface antigen playing an important role in the Wnt signaling pathway. ROR1 increases tumor cell proliferation, migration, invasion, and oncogenicity and triggers the formation of spheroids, the invasion of the extracellular matrix, or the development of tumor xenografts, which are functional features associated with CSC.	[29,30]
NES (Nestin)	Neuroepithelial stem cell protein	Intermediate filament protein involved in ne ovascularization and CSCs and closely related to vasculogenic mimicry formation.	[31]
TMSB4X (Tβ4)	Thymosin β4	A G-actin-sequestering peptide associated with the metastatic potential of tumor cells by stimulating cell migration. Tβ4 expression is strongly associated with CD133 expression and is characteristic of CSCs.	[32]

## Data Availability

Data presented in the study are available upon request from the corresponding author.
